# Neuronal mechanisms underlying activation of locomotor network by epidural electrical stimulation of the spinal cord

**DOI:** 10.1016/j.expneurol.2025.115187

**Published:** 2025-02-18

**Authors:** Pavel E. Musienko, Pavel V. Zelenin, Vladimir F. Lyalka, Polina Yu. Shkorbatova, Oleg V. Gorskii, Tatiana G. Deliagina

**Affiliations:** a Laboratory of Neuroprosthetics, Institute of Translational Biomedicine, St. Petersburg State University, 199034 St. Petersburg, Russia; b Pavlov Institute of Physiology, 199034 St. Petersburg, Russia; c Department of Neuroscience, Sirius University of Science and Technology, 354340 Sirius, Russia; d Federal Center of Brain Research and Neurotechnologies, 199330 Moscow, Russia; e Department of Neuroscience, Karolinska Institute, SE-17177 Stockholm, Sweden; f Life Improvement by Future Technologies Center, 143025 Moscow, Russia

**Keywords:** Epidural stimulation, Locomotion, Spinal neurons, Decerebrate cat

## Abstract

Epidural electrical stimulation of the spinal cord (ES) is used to restore/improve locomotor movements in patients. However, the neuronal mechanisms underlying activation of locomotor networks by ES are unknown. Here, we analyzed the effects of ES on the activity of individual spinal neurons of different functional locomotor groups. Neuronal activity was recorded in decerebrate cats before and during ES that evoked locomotion. During ES initial period (NW-period, before the locomotion onset), the activity was increased in most neurons as compared to that before ES. We found that ES-caused activation of neurons of locomotor network to the average level similar to that observed during locomotion is not sufficient for initiation of locomotion. We demonstrated that the onset of ES-evoked locomotion was associated with specific changes in the activity of individual neurons within functional locomotor groups and in their responses to epidural stimuli. With the locomotion onset, there was a trend for individual neurons with extremely high activity during NW-period to decrease their activity, while for neurons with extremely low activity to become more active. Also, in neurons contributing to generation of a specific mode of locomotion, peaks and troughs in responses to individual epidural stimuli were significantly less pronounced as compared with those observed during NW-period. We suggest that these changes allow ES to maintain the high level of excitability of the locomotor network necessary for its operation without distortion of the locomotor rhythm. The obtained results advance our understanding of the neuronal mechanisms underlying activation of locomotor network by ES.

## 1. Introduction

Locomotion is a vitally important motor function for both animals and humans. Epidural electrical stimulation of the spinal cord (ES) is used to restore/improve locomotor movements in patients (Dimitrijevic et al., 1998; Gerasimenko et al., 2001; Shapkova, 2004; Minassian et al., 2004; Minassia et al., 2007; Barthélemy et al., 2007). ES can evoke locomotion in animals with an intact spinal cord (Iwahara et al., 1992; Musienko et al., 2007, 2012) as well as in animals with spinal cord injury (Gerasimenko et al., 2003; Ichiyama et al., 2005; Lavrov et al., 2006; Courtine et al., 2009). It was suggested that ES activates the myelinated sensory fibers of the dorsal roots and dorsal column (Coburn and Sim, 1985; Rattay et al., 2003; Gerasimenko et al., 2006; Minassia et al., 2007; Capogrosso et al., 2013; Moraud et al., 2016). Since ES can evoke locomotion also in subjects with complete transection of the spinal cord (Gerasimenko et al., 2003; Barthélemy et al., 2007; Courtine et al., 2009), it was suggested that activated by ES afferents directly activate the spinal locomotor mechanisms. It was hypothesized that activation of afferents leads to gradual building up of neuronal activity in the locomotor network and, when it reaches a certain threshold, generation of locomotion starts (Grillner and Zangger, 1979). However, our knowledge about the neuronal mechanisms underlying activation of locomotor networks caused by ES is scarce.

EMG responses simultaneously evoked in multiple hindlimb muscles by individual pulses of ES applied with parameters optimal for inducing locomotor movements has been analyzed in subjects with intact and damaged spinal cord (Gerasimenko et al., 2003, 2005, 2006; Lavrov et al., 2006; Musienko et al., 2009). It was demonstrated that EMG responses to individual epidural stimuli contain earlier (presumably monosynaptic) and later (polysynaptic) components and both components are modulated in a phase-dependent manner during locomotion.

Previously, by recording the activity of the same individual spinal neurons during forward and backward locomotion evoked by ES, we revealed three functional groups of locomotor neurons. It was suggested that Group 1 generates the vertical component of steps (limb elevation and lowering) and when activated alone generates locomotion in place. Group 2 contributes to generation of both the vertical and horizontal (limb transfer from one extreme point to the other along the direction of locomotion) component of forward and backward steps, and Group 3 contains two sub-groups generating horizontal components for forward (Group 3a) and backward (Group 3b) steps (Musienko et al., 2012, 2022).

Here, first, to reveal contributions of different groups of locomotor neuron (Groups 1–3) as well as non-locomotor spinal neurons to ES-caused activation of the locomotor networks, the activity of individual neurons from these functional groups before ES and in the initial period of ES (before the onset of locomotion) was compared. Second, to reveal changes in the activity of these neurons associated with the onset of locomotion, the activity of individual neurons as well as their responses to individual epidural stimuli in the initial period of ES and during ES-caused forward and backward locomotion were compared. In the present study, a part of the database obtained in context of our previous study (Musienko et al., 2022) was used.

We found that ES-caused activation of neurons of the locomotor network to an average level similar to that observed during locomotion is not sufficient for initiation of locomotion. We demonstrated that the onset of ES-evoked locomotion is associated with specific changes in the activity of individual neurons within functional locomotor groups and in their responses to epidural stimuli. We suggest that these changes allow ES to maintain the high level of excitability of the locomotor network necessary for its operation without distortion of the locomotor rhythm.

## 2. Methods

### 2.1. Ethical approval

In the present study, a part of the database obtained in context of our previous study (Musienko et al., 2022) has been used. Experiments were carried out on four wild-type adult cats (*Felis catus*) of either sex (weighing 2.5–4.0 kg). The animals were obtained from the cat’s colony of Pavlov Institute of Physiology Animal House, where they were kept in cages with enriched environments and had free access to food and water.

All procedures were conducted in accordance with protocols (#01a/2017 and #01a/2018) approved by the Animal Care Committee of the Pavlov Institute of Physiology, St. Petersburg, Russia, and adhered to the European Community Council Directive (2010/63EU) and the guidelines of the National Institute of Health (*Guide for the Care and Use of Laboratory Animals*).

### 2.2. Surgical procedures

The surgical procedures are described in detail in the published study (Musienko et al., 2022). In short, the cats were deeply anesthetized with isoflurane (2–4 %) delivered in O_2_. The trachea was cannulated, and the carotid arteries were ligated. The animals were decerebrated at the precollicular-postmammillary level. A laminectomy was performed between the L4 and L7 segments for ES and recording of neurons. Bipolar electromyographic (EMG) electrodes were implanted bilaterally into *m. gastrocnemius lateralis* (Gast, ankle extensor) and *m. tibialis anterior* (Tib, ankle flexor). Anesthesia was discontinued after the surgical procedures. The experiments were started 2–3 h thereafter.

During the experiment, the rectal temperature and mean blood pressure of the animals were continuously monitored. The rectal temperature was maintained at 37 ± 0.5 °C with the help of heat irradiation. The blood pressure was kept at >80 mmHg. If needed, injection of prednisolone (3 mg/kg, i.m.) was done to stabilize the arterial pressure and to reduce brain swelling after decerebration.

### 2.3. Experimental design

The experimental design is described in detail earlier (Musienko et al., 2022). In short, the head, the vertebral column, and the pelvis of the decerebrate cat were fixed in a rigid frame. The forelimbs had no support, whereas the hindlimbs were positioned on the treadmill belt moving at the speed of 0.5 m/s either backward or forward in relation to the animal.

In each cat, locomotion was evoked by electrical epidural stimulation (ES) of the spinal cord. For this purpose, a ball electrode (d = 0.5 mm) was positioned on the dura mater close to the middle of the dorsal surface of the spinal cord at the L5-L6 level. The exact medio-lateral and rostro-caudal position was optimized to produce the highest quality of the locomotor movements in terms of stability, intra-limb coordination, and inter-limb symmetry. The following parameters of stimulation were used: biphasic square pulses; frequency, 5 Hz; pulse duration, 0.2–0.5 ms; current, 100–300 μA (Model 2100 Isolated Pulse Stimulator, A-M Systems, Sequim, WA, USA). Spinal neurons were recorded extracellularly from the spinal segment L4-L6 by means of commercially available multichannel electrode arrays (A1x32-10 mm-50-177; Neuronexus, Ann Arbor, MI). The array was inserted through small holes in the dura mater. The holes were made along the left-right extent of the gray matter. Within a hole, at the point of the insertion of the array, a small incision in the pia mater was made just sufficient for the spinal cord penetration. The array was inserted between blood vessels to prevent bleeding. We attempted to explore systematically the whole cross-section of gray matter except for the areas of motor nuclei. However, histological verification of the array track positions showed that while in L4 and L6 segments the array tracks positions covered the whole cross-section of the gray matter, in L5 spinal segment, all array tracks were located in the medial part of the gray matter.

**Fig. 1.**
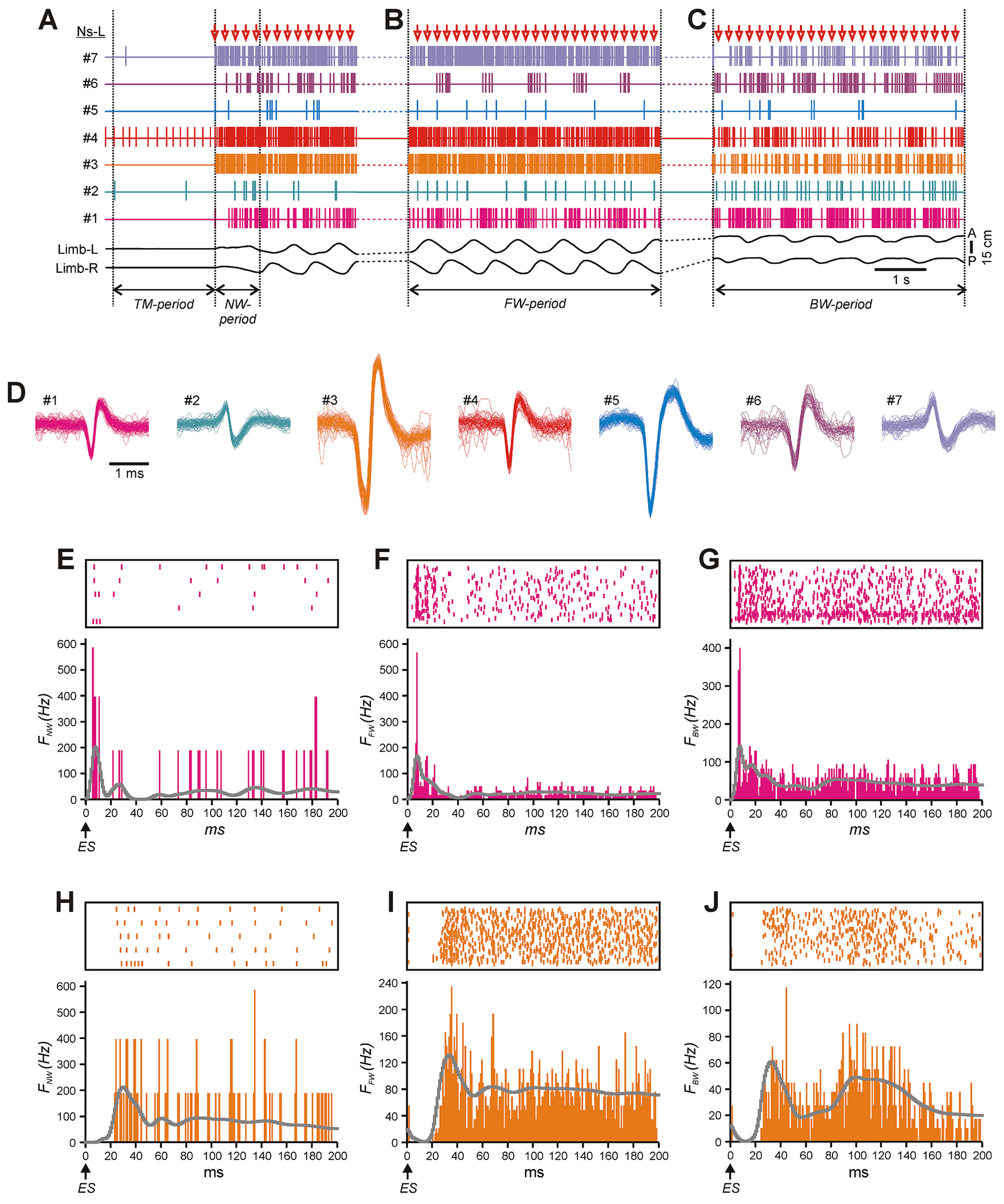
Activity of the same spinal neurons during TM-, NW-, FW-, and BW-period. A-D. Seven spinal neurons (##1–7) recorded simultaneously by the multichannel electrode array from the left side of the spinal cord during TM- and NW-period (A), FW- and BW-period (B and C, respectively), as well as overdrawn waveforms of their spikes extracted from the mass activity by spike sorting procedure (D). Neuronal activity was recorded along with movements of the left and right limbs (Limb L and Limb R). Individual epidural stimuli are indicated by red arrows. *E*-J. Rasters and the histograms of the ES-responses of the neuron #1 (E-G) and #3 (H-J) during NW-period (E,H), FW-period (F,I) and BW-period (G,J). Filtered histograms are shown in skyline mode by gray line. (For interpretation of the references to colour in this figure legend, the reader is referred to the web version of this article.)

We used only those neurons from the database obtained in our previous study (Musienko et al., 2022) that were recorded during the following procedure: first, backward motion of the treadmill belt was switched on (TM-period), then ES was switched on and evoked forward walking on the backward moving treadmill belt (FW-period), and, finally, the direction of the treadmill belt motion was reversed that led to backward walking (BW-period). The onset of ES did not result in immediate initiation of coordinated stepping. The period between the ES onset to the moment of generation of the first left-right alternating steps was defined as the period of “non-walking” (NW-period). Parameters of ES were chosen to be optimal for induction of locomotion and were not changed during recording. The neurons were recorded along with the EMG activity of hindlimb muscles and the anterior-posterior locomotor limb movements (recorded by means of two mechanical sensors; Limb R and Limb L in Fig. 1A).

To determine the approximate location of individual neurons (recorded at a definite position of the array by a particular electrode site) on the spinal cord cross-section, the lateral and vertical coordinates of the array tip were marked. The positions of the array in the spinal cord were verified by observation of the array tracks after histological procedure (see Musienko et al., 2022 for details). The positions of the recording sites were estimated in relation to the array track position.

The signals from the electrode array (neuronal activity), EMG electrodes, mechanical sensors were amplified and digitized with a sampling frequency of 24 kHz (neurons), 20 kHz (EMGs), and 5 kHz (sensors). Neuronal activity was band-pass filtered in the ranges 2.2–7500 Hz (Medusa preamplifiers, Tucker-Davis Technologies, Alachua, Florida, USA) and 300–6000 Hz (digital), EMGs were filtered only on the amplifier stage in the range 100–5000 Hz (Model 1700 Differential AC Amplifier, A-M Systems, Sequim, WA, USA). These signals, together with the signals indicating the switching-on and switching-off of the treadmill and ES, were recorded on a computer disk using the data acquisition system (RZ5 BioAmp Processor, Tucker-Davis Technologies, Alachua, Florida, USA).

### 2.4. Data analysis

The multiunit spike trains recorded by each electrode were separated into unitary waveforms representing the activity of individual neurons using the spike-sorting procedure in analysis software (Spike2, Cambridge Electronic Design, Cambridge, UK) (see Musienko et al., 2022 for details). An example of activity of seven neurons simultaneously recorded during backward treadmill motion in the absence of ES (“treadmill period”, TM-period) and during the initial period of ES that is before generation of the first alternating steps performed by the left and right hindlimbs (“no-walk period”, NW-period), during forward and backward walking (FW- and BW-period, respectively), as well as waveforms of their spikes are shown in Fig. 1, A, B, C and D, respectively. The activity of the neurons was typically modulated with the locomotor rhythm (Fig. 1B,C). As in our previous work (Musienko et al., 2022), the activity of a neuron was considered modulated if two necessary criteria were met: first, the mean burst frequency was significantly different from the mean interburst frequency (Student’s *t*-test, *P* < 0.05), and, second, the pattern of modulation in the majority of the locomotor cycles was consistent (the Pearson’s correlation coefficient between the profiles of activity in individual locomotor cycles and in the entire activity histogram was higher than 0.3 in more than 60 % of the locomotor cycles).

To reveal reaction of individual neurons to beginning of ES as well as a change in their activity caused by ES-evoked FW and BW, the mean frequencies of individual neurons during TM-, NW-, FW-, and BW-period were calculated.

To reveal responses in a hindlimb muscle to single epidural stimuli (mES-responses), its EMG was rectified and smoothed by moving average filters with a time constant of 50 ms, then the ES stimuli were used as triggers to average the processed EMG. Such ES-triggered averages were built separately for NW-, FW-, and BW-period.

To reveal responses of a neuron to single epidural stimuli (ES-responses), a post-stimulus histogram (PStH) with 200 ms duration and bin size 1 ms was generated. The number of spikes that fell in each bin was divided by the bin duration and by the number of stimuli, to produce the spiking frequency. The respective rasters and histograms for two neurons are shown in Fig. 1E–J. The high-frequency “noise” in PStHs was reduced by filtering with a Gaussian kernel. The filtered PStHs are shown in skyline mode by gray lines in Fig. 1E–J. The time constant (*T*) for the filtering was different for different bins: it was linearly dependent on the bin latency (*L*) (*T* = 0.05 * *L* + 2), thus for the 1st bin *T* was 2 ms, for the 200th (the last) bin *T* was 12 ms, and for the other bins *T* was between 2 and 12 ms. Such choice of the time constant allowed to preserve high time resolution for short-latency responses (though the random noise remained relatively high) and to suppress the noise in the long-latency responses (at the cost of time resolution). For each neuron, PStHs (ES-responses) for NW-, FW-, and BW-period were built separately.

To evaluate the degree of similarity between patterns of ES-responses of the same neuron at transition from NW- to FW-period and from FW- to BW-period, we calculated the coefficient of similarity (*K*_SIM_) that is the cosine similarity between a pair of histograms with subtracted background (the average frequency during the last 25 ms of the PStH) obtained in the corresponding conditions. Such analysis reveals covariations of the two functions, i.e., parallel changes of the instantaneous discharge frequency during the response, while dismissing differences in mean frequencies and depths of frequency change. So defined coefficient of similarity varies in a range from −1 (completely opposite changes of activity, e.g. excitation in one PStH and simultaneous inhibition of the same shape in the other PStH) to +1 (completely synchronous changes of activity in all aspects, that is latencies, shapes and durations). Examples of ES-responses of individual neurons recorded under three conditions and then compared using this method are shown in Fig. 8A–D.

To reveal typical components of ES-responses, non-negative matrix factorization (NNMF) of ES-response were computed using Scikit-learn data analysis package (Pedregosa et al., 2011). We considered 6 NNMF components, which were the minimal number of components sufficient to account on average for 95 % of ES-response shapes. Amplitudes of NNMF components were normalized so that a flat PStH with a constant firing frequency at 1 Hz would produce NNMF’s weighting coefficients of 1. NNMF was previously applied to analyses of EMG patterns (d’Avella et al., 2003; Ivanenko et al., 2005: Krouchev et al., 2006; Ting and Macpherson, 2005).

### 2.5. Statistical analyses

All quantitative data in this study are presented as mean ± SD. Paired Student’s *t*-test (two-tailed) was used for pairwise comparisons. Welch’s *t*-test (two-tailed) was used to characterize the statistical significance when comparing different means. To evaluate the statistical significance of difference in percentages of neurons with different patterns of responses to epidural stimuli in L4, L5 and in L6, as well as in different functional groups, we used Pearson’s χ^2^ test. *F*-test was used to compare SDs. The significance level for all tests was set at *P* = 0.05.

#### 2.5.1. Histological procedures

At the termination of the experiments, the cats were deeply anesthetized with isoflurane (5 %) and then perfused transcardially with isotonic saline followed by 4 % paraformaldehyde solution, which caused euthanasia. The L4 and L6 spinal segments were removed from the spine and stored in 20 % and 30 % sucrose until they sank. Then, regions of segments containing the recording sites were cut on a freezing microtome into 50 μm frontal sections. The sections were collected in 0.1 M PBS (pH 7.4) and then stained with cresyl violet. The positions of the array in the spinal cord were verified by observation of the array tracks. The positions of the recording sites were estimated in relation to the array track position.

## 3. Results

### 3.1. Motor responses to individual epidural stimuli

ES is necessary for both activation of the locomotor network and for maintenance of its excitability level necessary for generation of locomotor movements. We assumed that these processes could be reflected in motor (EMG) responses to individual epidural stimuli (mES-responses).

**Fig. 2.**
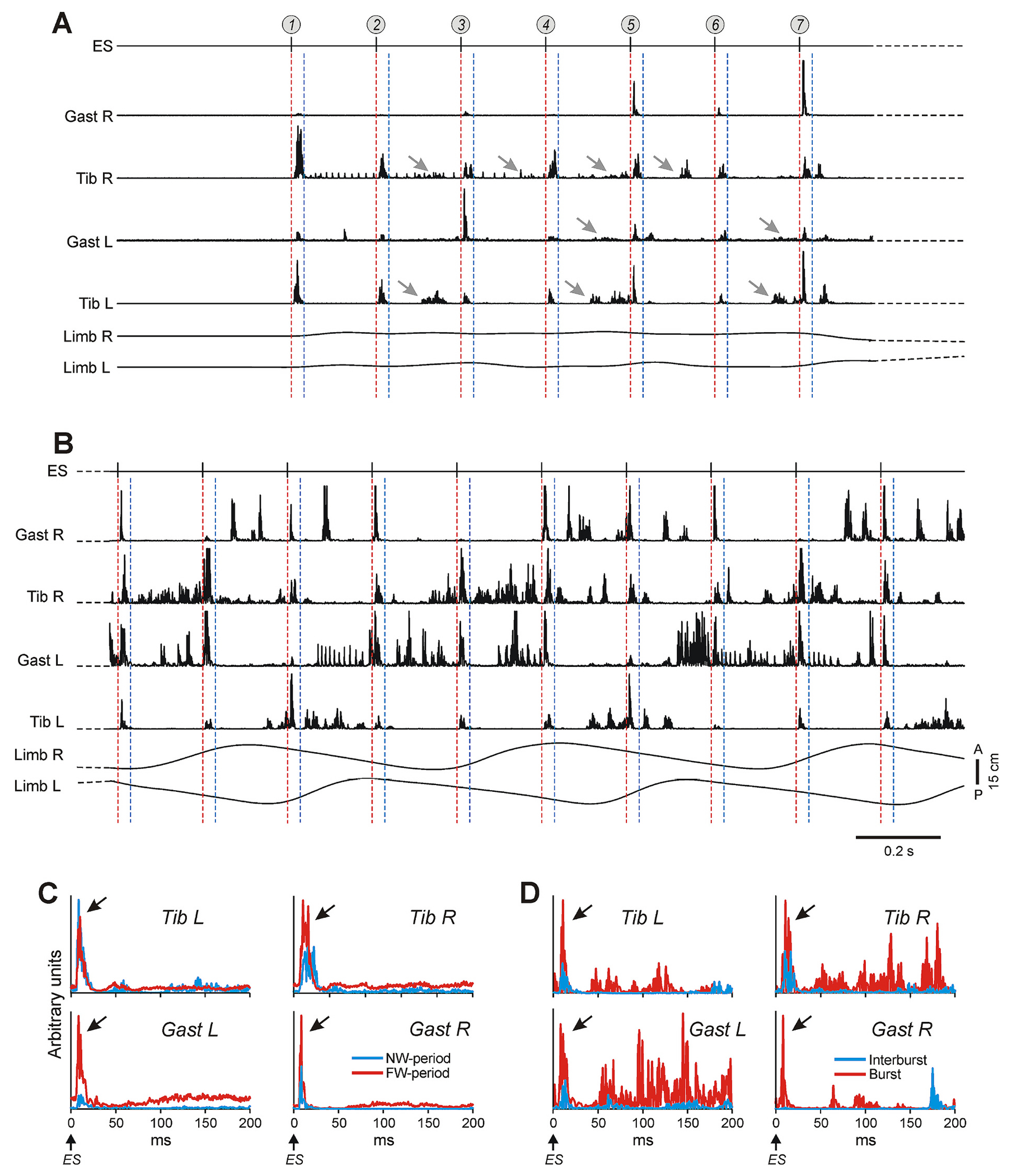
Responses of hindlimb muscles to ES stimuli during NW- and FW-period. A,B. EMGs of the right (R) and left (L) gastrocnemius and tibialis muscles (Gast R, Gast L and Tib R, Tib L) recorded along with movements of the right and left limbs (Limb R and Limb L) during NW-period (A) and FW-period (B). Red and blue dashed lines demarcate the first 30 ms after ES stimuli that contain the earlier component of mES-response. Gray arrows mark the later component of the mES-responses. In A, numbers in circles indicate the first seven epidural stimuli (ES) during NW-period. C. Comparison of averaged mES-responses in hindlimb muscles during NW-period (blue line) and FW-period (red line). D. Comparison of averaged mES-responses in hindlimb muscles during their bursts (red line) and interbursts (blue line) in FW-period. In C and D, average over 7 mES-responses. In C,D, black arrows indicate the earlier component of the mES-responses. (For interpretation of the references to colour in this figure legend, the reader is referred to the web version of this article.)

A representative example of mES-responses in flexor and extensor muscles in initial period of ES (NW-period) and during well-coordinated forward walking (FW-period) is shown in Fig. 2, A and B, respectively. During NW-period (Fig. 2A), one can distinguish two components in mES-response, the earlier short latency (6.9 ms - 8.2 ms) and short duration (8.2 ms – 21.9 ms) component (between red and blue dashed lines) as well as the later long latency (79.5 ms – 142.1 ms) and long duration (30.1 ms – 122.8 ms) component (marked by gray arrows). The earlier component was consistently observed after each epidural stimulus in all muscles (Fig. 2A). However, on average, it was substantially stronger in ankle flexor muscles (Tib L and Tib R) as compared with that in ankle extensors (Gast L and Gast R) (blue responses in Fig. 2C). Most likely, it can be explained by a higher level of excitability of flexor motoneurons caused by activation of flexors’ stretch receptor afferents due to limb extension produced by treadmill belt motion. The later component was less consistent (e.g., in Tib L, it was absent in response to epidural stimuli #1, #3, and #5; Fig. 2A) and more consistently expressed in flexors than in extensors (e.g., it was absent in Gast R; Fig. 2A). The amplitude of both earlier and later responses fluctuated independently. Thus, in Gast L, the earlier component of the mES-response to epidural stimulus #3 was large, but the later component was absent, while the earlier component of the mES-response to epidural stimulus #4 was small but the later response was present. One can assume that already during NW-period, ES activated some parts of locomotor network which can modulate independently two components of the mES-response.

As compared with NW-period, during FW (Fig. 2B) and BW (not illustrated), on average, the amplitude of the earlier component of the mES-response (indicated by black arrows in Fig. 2C,D) was substantially increased in extensor muscles but not always in flexor muscles (Fig. 2C). The amplitude of the earlier component of the mES-response was modulated in the locomotor rhythm in both flexor and extensor muscles. It was enhanced during locomotor EMG burst and dramatically reduced in the interburst period (Fig. 2D). The presence of the later component in the mES-response during locomotion is not clear, since it was impossible to extract it from the burst activity because of large fluctuation in its latency and duration.

The general features of the mES-responses in flexor and extensor muscles were similar in all studied cats; they were also similar to those described in earlier studies (Gerasimenko et al., 2003, 2005, 2006; Lavrov et al., 2006; Musienko et al., 2009).

### 3.2. Characterization of neuronal database

Altogether, the activity of 315 individual spinal neurons (from the database of our previous study, Musienko et al., 2022) recorded in the four cats during sequential TM-, NW-, FW-, and BW-periods (see Materials and Methods for the details) have been used for the analysis (Table 1). They included 56, 62, and 197 neurons recorded in the L4, L5 and L6 spinal segments, respectively. Most neurons (~88 % from L4, ~84 % from L5, and ~ 74 % from L6) were active and modulated during FW and/or BW (see criteria for modulation in Musienko et al., 2022). Modulated neurons belonged to different functional locomotor groups (Table 1) revealed in our previous study (Musienko et al., 2022). It was suggested that Group 1 generates a vertical component of the step, Group 3 belongs to the networks determining the forward (Group 3a) and backward (Group 3b) direction of stepping, and Group 2 contributes to generation of both the vertical and horizontal components of the step.

### 3.3. Effects of ES on activity of spinal neurons

#### 3.3.1. Effects of the onset of ES on activity of spinal neurons

**Fig. 3.**
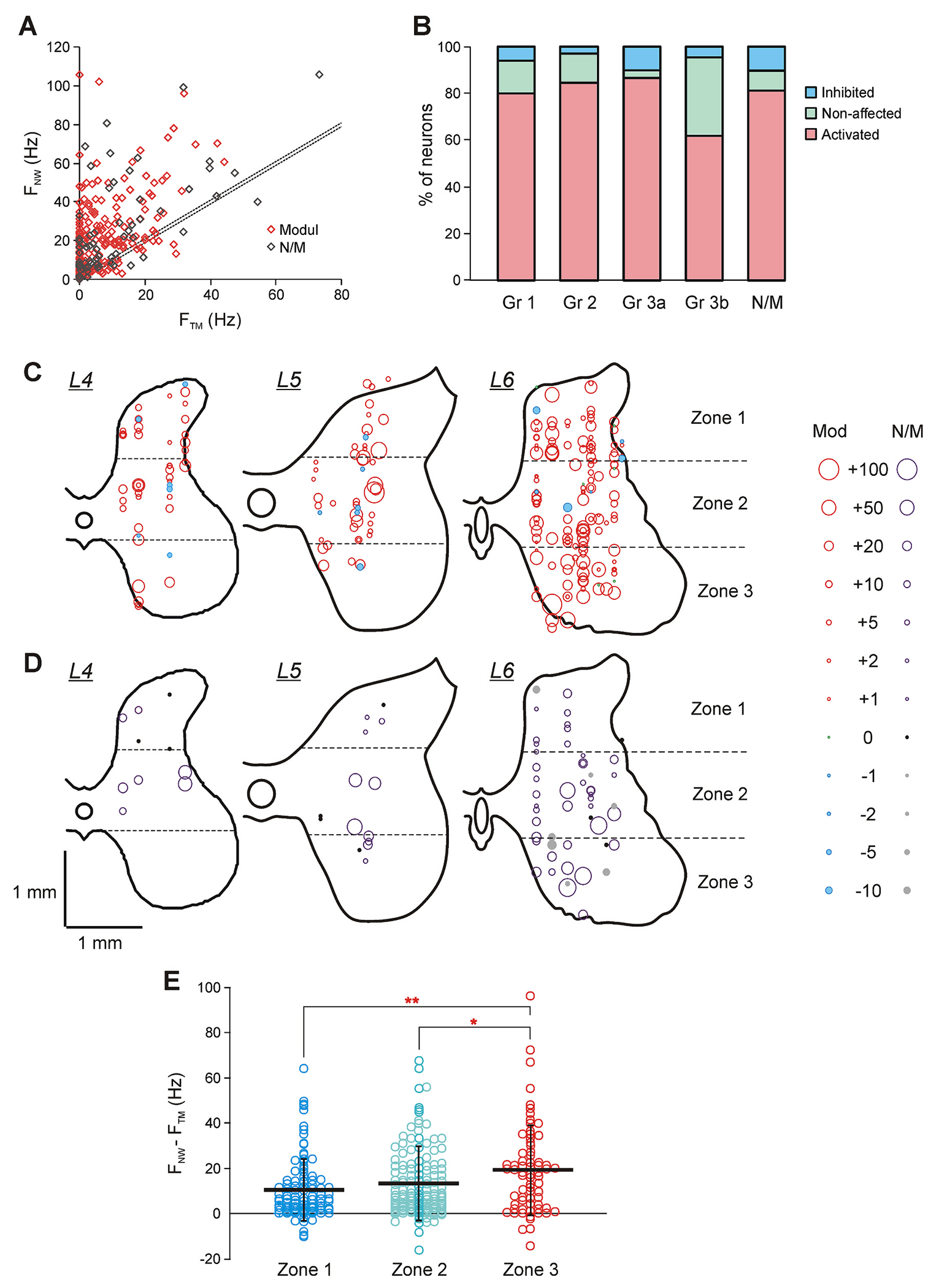
Changes in the activity of spinal neurons with the onset of ES (with transition from TM-period to NW-period). A. The mean frequency of individual modulated (Modul) and non-modulated (N/M) neurons during NW-period (F_NW_) and TM-period (F_TM_). Dotted lines delineate neurons with a change in the mean frequency less or equal to 1 Hz. B. Relative number of neurons activated, non-affected, and inhibited by the onset of ES in different functional groups of neurons. The number of neurons in Gr 1, Gr 2, Gr 3a, Gr 3b, and N/M is *n* = 101, 65, 60, 21, and 68, respectively. C,D. Positions of modulated (C) and non-modulated (D) neurons with different effects of the ES onset on their activity on the cross-section of the spinal cord, recorded in L4 (*N* = 1, *n* = 56), L5 (*N* = 1, *n* = 62) and L6 (*N* = 2, *n* = 197) segments. E. The mean and SD values of the frequency change with the onset of ES (F_NW_ – F_TM_) in sub-populations of neurons located in zones 1, 2, and 3 of the gray matter (*N* = 4, *n* = 100, 146, and 69). Significance: zone 3 vs zone 1 and zone 2, unpaired *t*-test, respectively, *P* = 0.002 and *P* = 0.03. Indication of significance level: * *P* < 0.05, ** *P* < 0.01.

To reveal neurons that can potentially contribute to activation of the locomotor network caused by ES, we analyzed effects of switching on of ES on the activity of individual spinal neurons. Fig. 3A shows the mean frequency of individual modulated and non-modulated neurons during TM-period plotted against their mean frequency during NW-period. In the overwhelming majority of neurons (79 %) the mean frequency was increased (the diamonds above the area delineated by dotted lines, diagonals y = x ± 1 Hz in *A*). However, in some neurons (15 %) the mean frequency was almost not changed (the diamonds within the area delineated by dotted diagonals y = x ± 1 Hz) and in some neurons (6 %), it was decreased (the diamonds below the area delineated by dotted diagonals y = x ± 1 Hz). Each of three populations of neurons (activated, inhibited and non-affected by ES) contained both modulated neurons (that belonged to functional locomotor Groups 1–3) and non-modulated neurons (Fig. 3B). Proportions of activated, inhibited and non-affected by ES neurons were rather similar in locomotor groups contributing to control of both FW and BW (Groups 1 and 2), to control of FW only (Group 3a), and in non-modulated neurons although some difference between proportions of non-affected by ES neurons in Group 1 and Group 3a was statistically significant (14 % and 3 %, respectively; χ^2^ test, *P* = 0.03). In Group 3b, contributing to control of BW only, the proportion of non-affected by ES neurons (33 %) was significantly higher than that in any of other groups (14 % in Group 1, 12 % in Group 2, 3 % in Group 3a, 9 % in non-modulated neurons; χ^2^ test, *P* = 0.03, *P* = 0.03, *P* = 2 × 10^−4^, *P* = 5 × 10^−3^, respectively). Also, the proportion of activated by ES neurons in Group 3b was substantially lower than those in any of other groups. The differences with Group 2 and Group 3a were statistically significant (62 % in Group 3b vs 85 % in Group 2 and vs 87 % in Group 3a; χ^2^ test, *P* = 0.03 and *P* = 0.01, respectively). This could be explained by the low level of excitability of Group 3b neurons reflected in their significantly lower than in other Groups level of spontaneous activity (Musienko et al., 2022), as well as the level of activity during TM-period: 2.7 ± 3.5 Hz in Group 3b vs 5.0 ± 7.7 Hz in Group 1, 6.6 ± 9.5 Hz in Group 2, 7.3 ± 10.0 Hz in Group 3a, 11.1 ± 14.9 Hz in non-modulated neurons (unpaired *t*-test, *P* = 0.032, *P* = 6 × 10^−3^, *P* = 2.6 × 10^−3^, *P* = 4.2 × 10^−5^, respectively).

Fig. 3C,D shows the location of individual modulated (*C*) and non-modulated (*D*) neurons activated, inhibited as well as not affected by the ES onset. One can see that activated, inhibited, and not affected neurons were found in each of L4-L6 segments, in different areas of gray matter and intermixed. Also, proportions of activated, inhibited, and non-affected by ES neurons were similar in L4, L5, and L6 segments (respectively, 79 %, 9 %, and 13 % in L4; 71 %, 6 %, and 23 % in L5; 82 %, 5 %, and 13 % in L6; χ^2^ test, *P* = 0.27).

To characterize quantitatively the activity change caused by the ES onset, the difference between the mean frequency during NW- and TM-period of individual neurons was averaged separately over neurons in each of locomotor Groups 1–3 as well as over non-modulated neurons, over the entire population of neurons in each of L4-L6 segments, and over neurons in each of three zones of the gray matter (zone 1 corresponds to the dorsal part of the dorsal horn, zone 3 – to the ventral horn, and zone 2 – to the intermediate area of the gray matter, Fig. 3E). On average, a significant increase in the firing frequency by 10.1–14.6 Hz for different segments and by 9.2–15.3 Hz for different functional groups of spinal neurons (Groups 1–3 and non-modulated neurons) was observed (Table 2). The difference in the mean frequency increase between any two segments, as well as between any functional groups of neurons was absent (unpaired *t*-test, *P* > 0.06 and *P* > 0.18, respectively). Also, on average, the changes in the activity of neurons located in zones 1 and 2 were similar (Fig. 3E). By contrast, neurons located in zone 3 exhibited a significantly higher increase in activity as compared with those located in zone 1 and zone 2 (unpaired *t*-test, *P* = 0.002 and *P* = 0.03, respectively; Fig. 3E).

#### 3.3.2. Changes in activity of spinal neurons with transition from NW- to FW- and BW-period

**Fig. 4.**
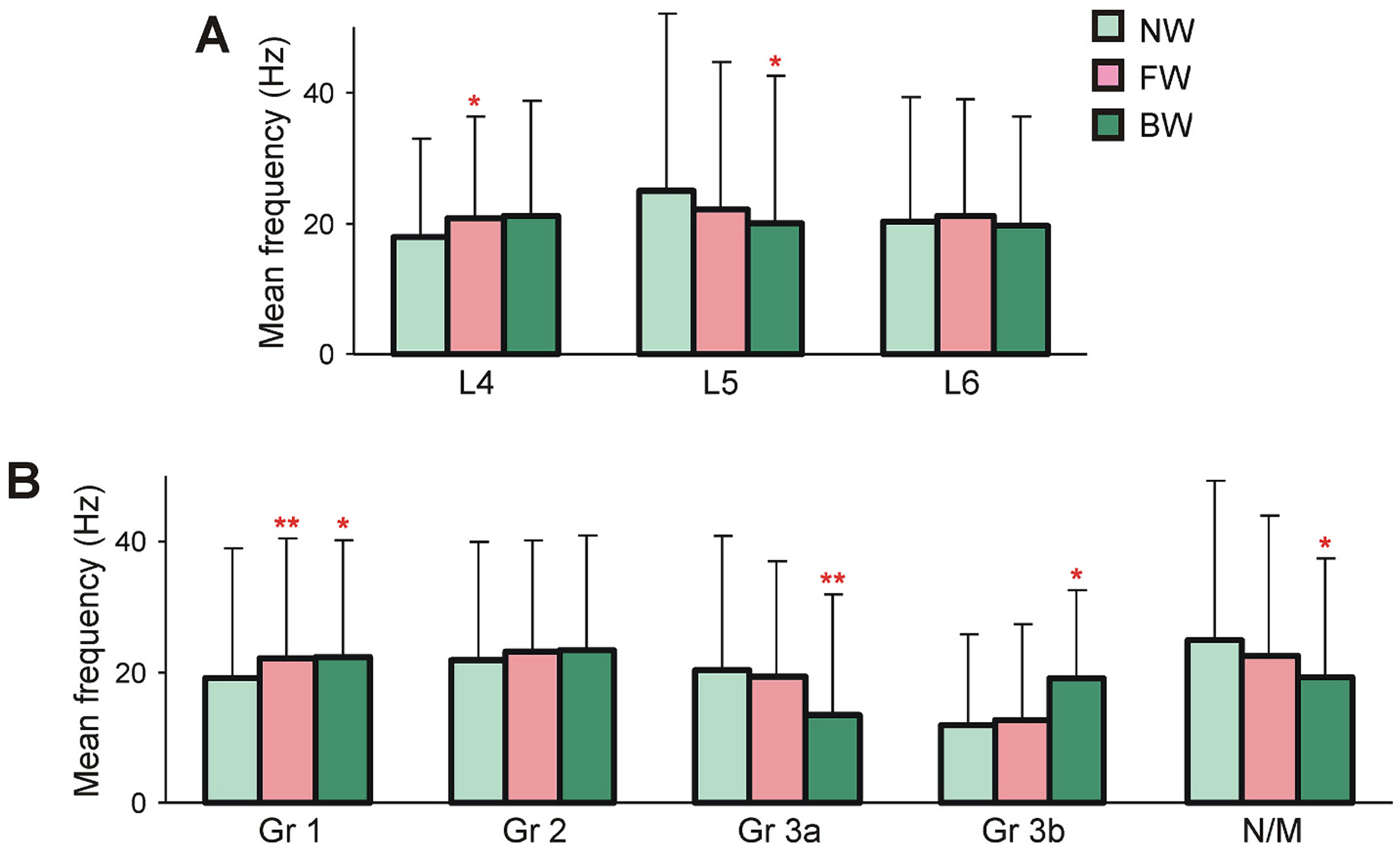
Changes in the population activity of spinal neurons with the onset of locomotion. A. Comparison of the mean frequencies during NW-, FW-, and BW-period in neurons of L4 (*n* = 56), L5 (*n* = 62) and L6 (*n* = 197) segments. B. Comparison of the mean frequencies during NW-, FW-, and BW-period in different functional groups of neurons (Group 1: *n* = 101; Group 2: *n* = 65; Group 3a: *n* = 60; Group 3b: *n* = 21; non-modulated neurons: *n* = 68). Red asterisks indicate a significant difference in the mean frequency during FW- or BW-period as compared to NW-period for individual segments or for individual functional groups of neurons. Indication of significance level: * *P* < 0.05; ** *P* < 0.01. (For interpretation of the references to colour in this figure legend, the reader is referred to the web version of this article.)

To examine whether initiation of locomotion is associated with a change in the population activity of neurons residing in specific spinal segments, we compared neuronal activity in segments L4, L5, L6 during NW-, FW-, and BW-period (Fig. 4A). We found that in each of three segments the activity level was similar in NW-, FW-, and BW-periods although a small increase in the mean frequency during FW in segment L4 and a small decrease during BW in segment L5 were statistically significant (paired *t*-test; *P* = 0.01 and *P* = 0.02, respectively).

To examine whether initiation of locomotion is associated with a change in the level of population activity of specific functional groups, we compared their mean frequency during NW-, FW-, and BW-period (Fig. 4B). We found that in Groups 1 and 2 (that contribute to generation of both FW and BW locomotion) as well as in non-modulated neurons, the population activity was similar in NW-, FW-, and BW-period although a small increase in the mean frequency during FW- and BW-period in Group 1 and some decrease in the activity of non-modulated neurons during BW as compared to those during NW-period were statistically significant (paired *t*-test; *P* = 6 × 10^−3^, *P* = 0.03, and *P* = 0.02, respectively). In Groups 3a and 3b (that contribute, respectively, to generation of FW only and to generation of BW only), the values of the mean frequency during NW- and FW-period were similar although they were lower in Group 3b than in Group 3a. However, during BW-period the activity significantly decreased in Group 3a and increased in Group 3b as compared with the corresponding values during NW-period (paired *t*-test; *P* = 3 × 10^−3^ and *P* = 0.03, respectively). Groups 3a and 3b belong to the part of the locomotor network which is driven by sensory feedback from the limb (Musienko et al., 2012, 2022). Most likely, backward displacement of limbs caused by the treadmill motion during NW-period, as well as during stance of FW inhibits Group 3b neurons which results in their low general activity. Correspondingly, forward displacement of limbs during stance of BW activates Group 3b and inhibits Group 3a neurons. To conclude, the obtained results demonstrate that initiation of locomotion is associated neither with substantial changes in population activity of specific functional groups of neurons nor with substantial changes in the population activity of neurons located in each of L4-L6 segments.

**Fig. 5.**
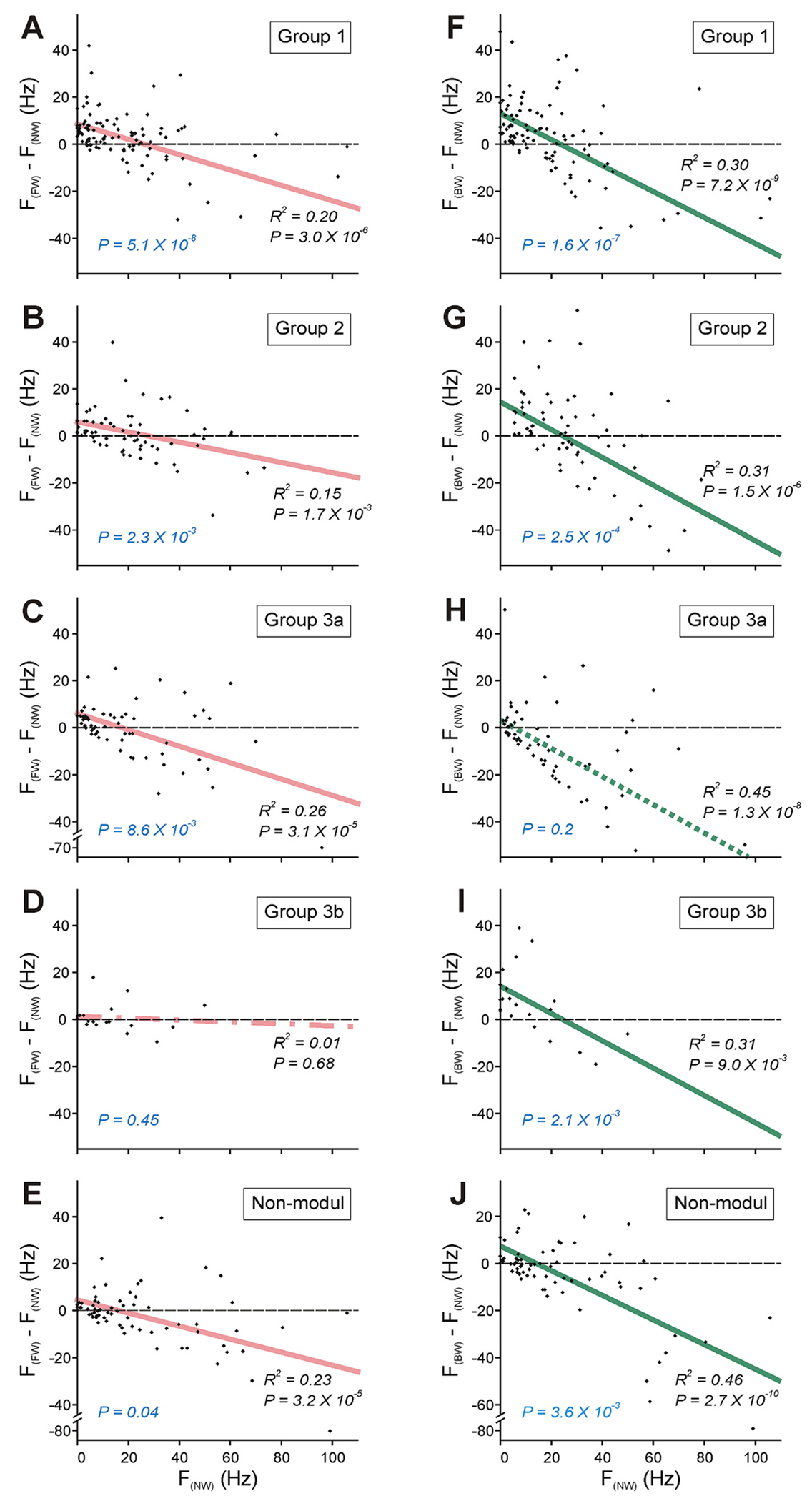
Changes in the activity of individual spinal neurons with the onset of locomotion. A-J. Correlation of the mean frequency of individual neurons of different functional groups during NW-period (*F_(NW)_*) with the change in their mean frequency during FW-period (*F_(FW)_* - *F_(NW)_*; A-E) and with the change in their mean frequency during BW-period (*F_(BW)_* - *F_(NW)_*; *F-J*). Pink and green lines present the linear regressions for transitions from NW- to FW-period and from NW- to BW-period, respectively. Significance (*P* values) of the negative correlation and the positive *y*-intercept of the linear regression are indicated in black and blue, respectively. Regression line is solid if the negative correlation and the positive *y*-intercept are significant, dotted if the negative correlation is significant while the positive *y*-intercept is not significant, dash-dotted if both the negative correlation and the positive *y*-intercept are not significant. (For interpretation of the references to colour in this figure legend, the reader is referred to the web version of this article.)

During transition from NW- to FW- or BW-period, the activity of individual neurons of the same functional group could change differently. To reveal possible trends in the change of the activity of individual neurons with the onset of locomotion in different functional groups, the mean frequency of individual neurons of a particular group during NW-period (*F_(NW)_*) was plotted against their change in the mean frequency during transition from NW- to FW-period (*F_(FW)_* - *F_(NW)_*, Fig. 5A–E) or during transition from NW- to BW-period (*F_(BW)_* - *F_(NW)_*, Fig. 5F–J). We found a significant negative correlation between plotted parameters for both transition from NW- to FW-period and transition from NW- to BW-period for Group 1 and Group 2 neurons (which contribute to generation of both FW and BW) (black *P* values in Fig. 5A–E). Also, the positive intercept level of corresponding regression lines with the ordinate axis was significant (blue *P* values in Fig. 5A–E). Thus, within Group 1 and Group 2 populations, the neurons with the lower activity during NW-period (the neurons located in the area to the left from the point of intersection of the regression line with the abscissa) exhibited tendency to the activity increase, while the neurons with the higher activity during NW-period (the neurons located in the area to the right from the point of intersection of the regression line with the abscissa) exhibited tendency to the activity decrease during FW and BW.

In contrast to Groups 1 and 2, within Group 3a and Group 3b populations (that contribute to generation of FW only and BW only, respectively), such tendency was revealed only for transition from NW-period to the mode of locomotion to which generation the Group contributed. Thus, both the negative correlation and the positive intercept level of the regression line with the ordinate axis were significant only during transition from NW- to FW-period for Group 3a neurons (Fig. 5C) and only during transition from NW- to BW-period for Group 3b neurons (Fig. 5I). By contrast, in Group 3a population, the neurons with any level of activity during NW-period exhibited tendency to the activity decrease during BW-period (the negative correlation was significant but the intercept level of the regression line with the ordinate axis did not differ from 0, Fig. 5H), and in Group 3b population, any tendency in the activity change of individual neurons during transition from NW-to FW-period was absent (the negative correlation was very weak and insignificant, Fig. 5D). Surprisingly, in population of non-modulated neurons, the tendency in a change of the activity of individual neurons with transition from NW-period to locomotion was similar to that observed in populations of Group 1 and Group 2 neurons (both the negative correlation and the positive intercept level of the regression line with the ordinate axis were significant for transition from NW- to both FW- and BW-period, Fig. 5E,J).

### 3.4. Responses of spinal neurons to individual epidural stimuli (ES-responses)

#### 3.4.1. Patterns of ES-responses of individual neurons in NW-period

**Fig. 6.**
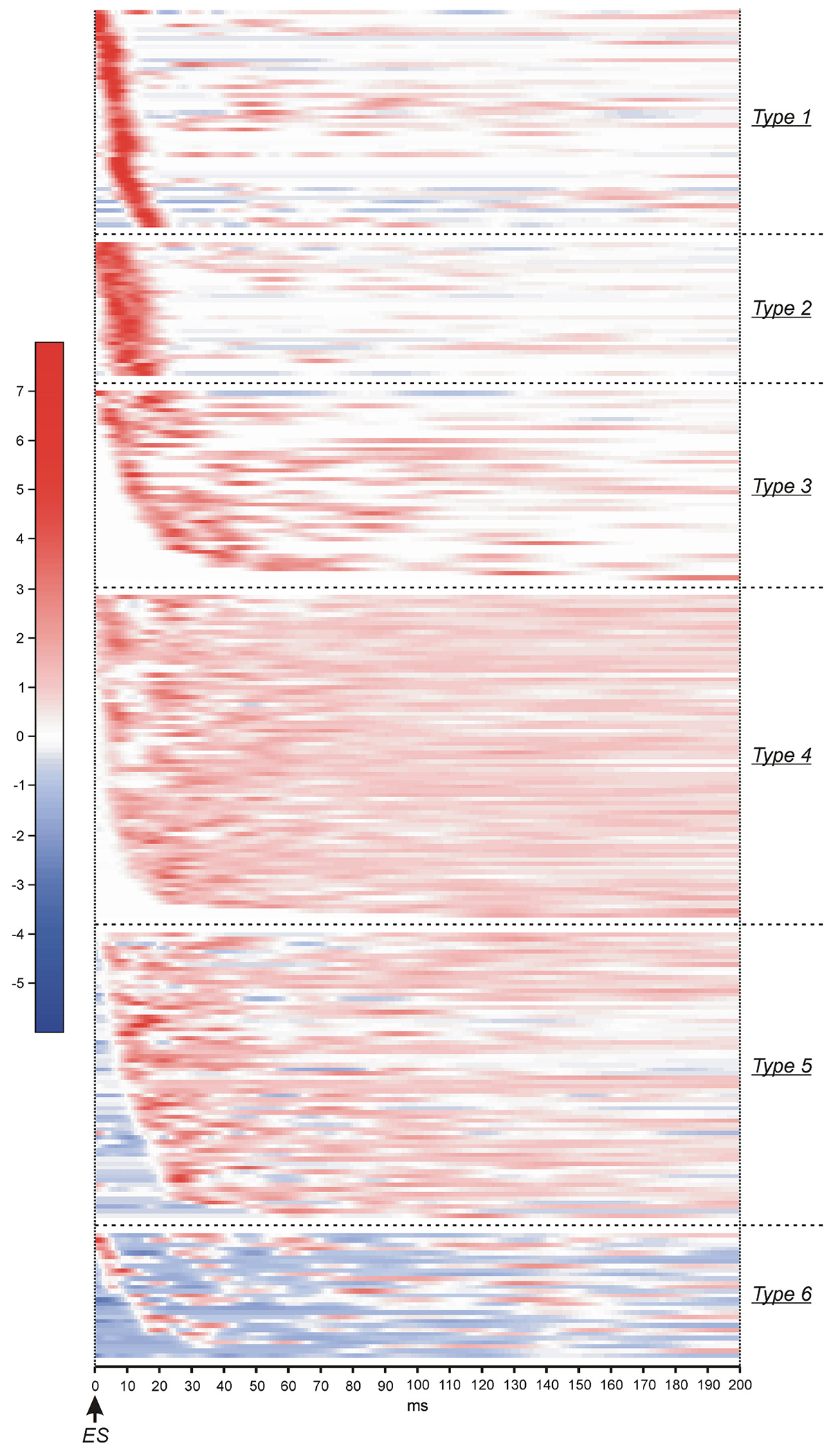
Patterns of ES-responses of individual neurons during NW-period. Patterns are presented as heatmaps (see Results for details). Different types of patterns are demarcated by dotted lines. Number of animals and number of neurons, *N* = 4 and *n* = 295, respectively (20 neurons that were inactive during both TM- and NW-periods are not shown).

The overwhelming majority (94 %) of spinal neurons responded to individual epidural stimuli. To reveal patterns of ES-responses of individual neurons in NW-period, their mean frequency before switching on ES (in TM-period) was subtracted from their histograms obtained for NW-period, the result was normalized to the root mean square to get rid of the absolute values and consider only response profiles, and then the heatmaps of the ES-responses were generated (Fig. 6). All patterns of ES-responses can be divided into six types. Type 1 and Type 2 ES-patterns were characterized by strong and relatively short earlier excitatory response. Type 1 ES-patterns had shorter duration of the response than Type 2 (~10 ms in Type 1 and ~ 20 ms in Type 2) and could appear with longer latency (up to ~20 ms in Type 1 and up to ~10 ms in Type 2). Type 3 ES-patterns were characterized by sequential waves of excitation started in individual neurons with different latencies up to ~50 ms. Types 4 and 5 ES-patterns were characterized by long lasting excitation that continues until the next epidural stimuli. In Type 5, the excitation was preceded by inhibitory response, while in Type 4 it was preceded by activity which did not differ from activity in TM-period. However, since in neurons with Type 4 ES-patterns the excitation continued until the next ES stimulus, one can expect that the return to the level of activity in TM-period observed just after ES stimulus is also caused by the inhibition. Finally, Type 6 patterns were characterized by predominating inhibition.

**Fig. 7.**
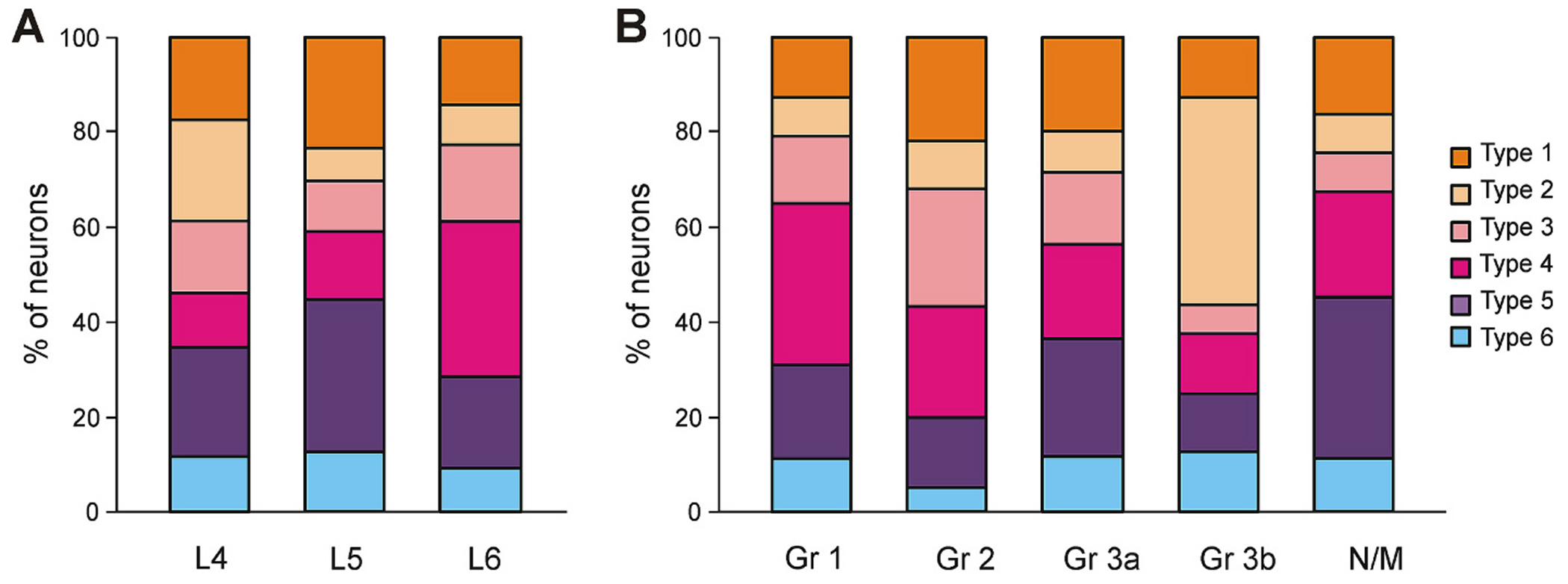
Percentage of neurons with different Types of ES-response patterns during NW-period in L4-L6 segments and within different functional groups of spinal neurons. A. Comparison of the relative number of neurons with different Types of ES-response in L4 (*N* = 1, *n* = 52), L5 (*N* = 1, *n* = 56), and L6 (*N* = 2, *n* = 187). B. Comparison of the relative number of neurons with different Types of ES-response in different functional groups of neurons. Group 1: *n* = 97; Group 2: *n* = 60; Group 3a: *n* = 60; Group 3b: *n* = 16; non-modulated neurons: *n* = 62. Abbreviations as in Fig. 3B.

Neurons with Types 1–6 patterns of ES-responses were found in each of L4-L6 segments (Fig. 7A). However, the proportion of neurons with Type 2 pattern was more than two fold higher in L4 segment (21 %) as compared to those in L5 (7 %) and L6 (9 %) (χ^2^ test, *P* = 0.035 and *P* = 0.010, respectively), while the proportion of neurons with Type 4 pattern was more than two fold higher in L6 (33 %) as compared to those in L4 (11 %) and L5 (14 %) (χ^2^ test, *P* = 4 × 10^−3^ and *P* = 9 × 10^−3^, respectively). Finally, the proportion of neurons with Type 5 pattern was substantially higher in L5 as compared to those in L4 and L6 segments. The difference between L5 (32 %) and L6 (19 %) was significant (χ^2^ test, *P* = 0.04). The proportions of neurons with Type 1, Type 3, and Type 6 patterns were similar in L4-L6 segments (χ^2^ test, *P* ≥ 0.30).

Similarly, each of locomotor groups of neurons (Groups 1–3) as well as the group of non-modulated neurons contained neurons with all six Types of patterns of ES-responses (Fig. 7B). Proportions of neurons with different types of patterns were similar in Groups 1, 2, 3a, and non-modulated neurons (χ^2^ test, *P* ≥ 0.16), but significantly differed from those in Group 3b (χ^2^ test, *P* ≤ 0.03). In particular, in Group 3b, the proportion of neurons with Type 2 pattern of ES-responses was dramatically higher than those in other locomotor groups as well as in non-modulated neurons (χ^2^ test: 44 % in Group 3b vs 8 % in Group 1, *P* = 10^−4^; 44 % in Group 3b vs 10 % in Group 2, *P* = 10^−3^; 44 % in Group 3b vs 8 % in Group 3a, *P* = 6 × 10^−4^; 44 % in Group 3b vs 8 % in non-modulated neurons, *P* = 4 × 10^−4^).

#### 3.4.2. Comparison of ES-responses of individual neurons in NW-, FW-, and BW-period

**Fig. 8.**
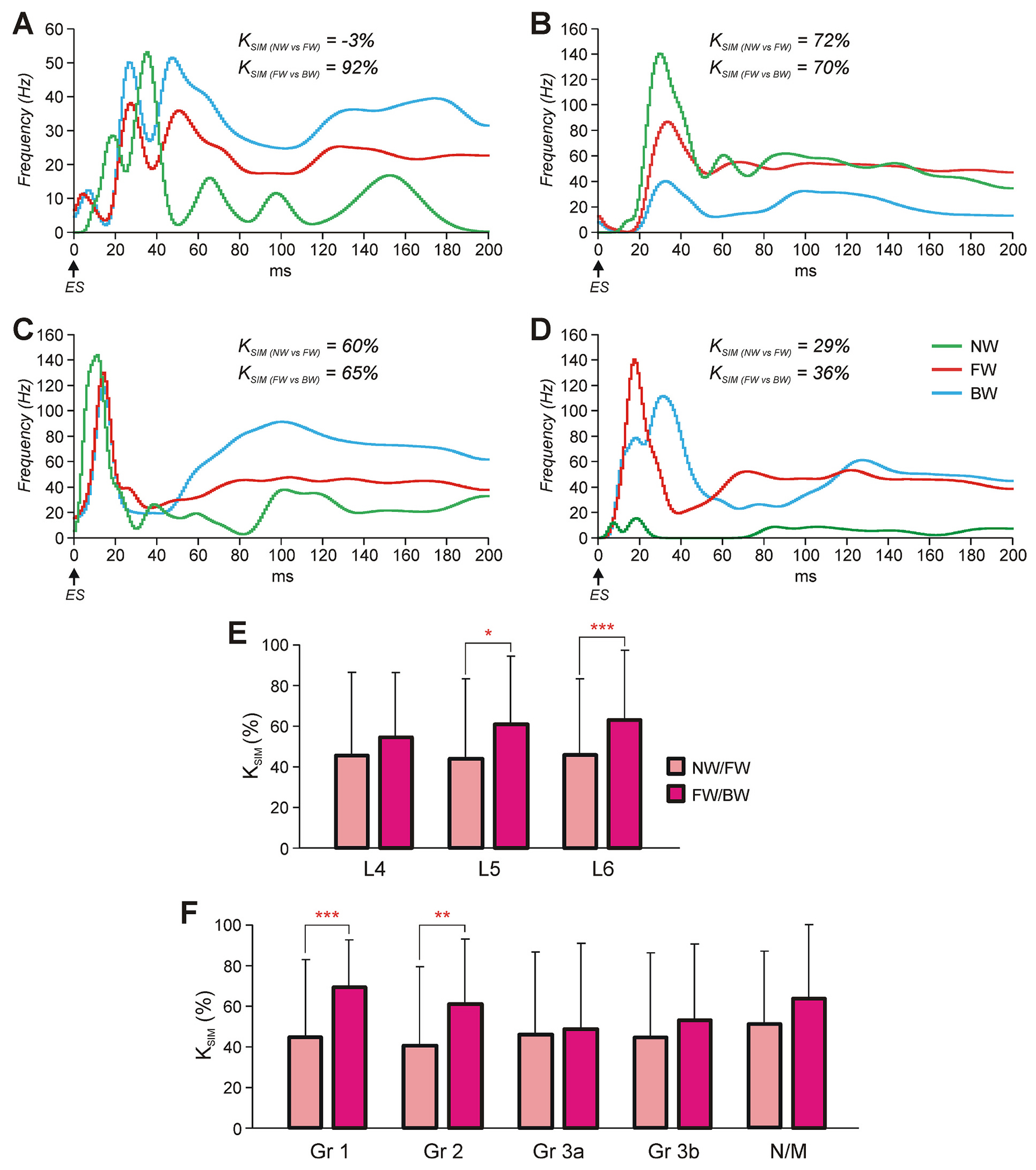
Comparison of ES-responses of individual neurons during NW-, FW-, and BW-period. A-D. Examples of ES-responses of four neurons (A, B, C, and D) during NW-, FW-, and BW-period. The coefficient of similarity, that is the correlation coefficient between the ES-response histograms obtained under two conditions (NW- and FW-period, *K*__SIM (NW_ vs _FW)__; FW- and BW-period, *K*_*SIM (FW* vs *BW)*_), is indicated. Neurons were recorded in L6 segment and belonged to following functional groups: Group 1 (A), Group 2 (B,C), and non-modulated (D). E,F. Comparison of the mean ± SD values of *K*_*SIM (NW* vs *FW*_) and *K*_*SIM (FW* vs *BW)*_ in populations of neurons recorded in L4, L5, and L6 (E), as well as in different functional groups (F). Number of animals and number of neurons in E and F are the same as in Fig. 7, A and B, respectively.

To evaluate the degree of similarity between patterns of ES-responses of the same neuron during NW- and FW-period, as well as between FW- and BW-period, we calculated their corresponding coefficients of similarity that revealed parallel changes of the instantaneous discharge frequency during the response, while dismissing differences in mean frequencies and depths of frequency change. (*K*_SIM_, see Materials and Methods). Fig. 8A–D shows the histograms of ES-responses during NW-, FW, and BW-period for four individual neurons with their coefficients of similarity. One of them had substantially different patterns of ES-response during NW- and FW-period (compare green and red histograms in Fig. 8A), but almost the same pattern during FW- and BW-periods (compare red and blue histograms in Fig. 8A) and, respectively, low and high values of the corresponding coefficients of similarity (*K_SIM (NW_* vs *_FW)_* = −3 % and *K_SIM (FW_* vs *_BW)_* = 92 %). Another one had not the same but still rather similar patterns of responses under all three conditions (compare green, red, and blue histograms in Fig. 8B), and correspondingly, slightly lower coefficients of similarity (*K_SIM (NW_* vs *_FW)_* = 72 % and *K_SIM (FW_* vs *_BW)_* = 70 %). The third one had less similar patterns of responses under all three conditions (compare green, red, and blue histograms in Fig. 8C), and correspondingly, lower coefficients of similarity (*K_SIM (NW_* vs *_FW)_* = 60 % and *K*_*SIM (FW* vs *BW)*_ = 65 %). Finally, the neurons shown in Fig. 8D had substantially different patterns of ES-responses during NW- and FW- periods as well as during FW- and BW -periods, and, correspondingly, low values of coefficients of similarity (*K*_*SIM (NW* vs *FW)*_ = 29 % and *K*_*SIM (FW* vs *BW)*_ = 36 %).

Fig. 8E,F shows the mean values of the coefficient of similarity between ES-response patterns during NW- and FW-period as well as during FW- and BW-period in each of L4-L6 segments (*E*), and in each of functional groups of neurons (*F*). One can see that the mean values of the coefficient of similarity during NW- and FW-period in L4, L5, and L6 were similar (about 45 %, *E*). In L5 and L6, the mean value of the coefficient of similarity was significantly higher during FW- and BW-period, than that during NW- and FW-period (paired *t*-test: 61 ± 34 % vs 44 ± 39 %, *P* = 0.01 and 63 ± 34 % vs 46 ± 38 %, *P* = 2.6 × 10^−6^, respectively), while in L4 segment, the difference was insignificant (paired *t*-test: 55 ± 36 % vs 46 ± 41 %, *P* = 0.21).

In groups of locomotor neurons, which contribute to control of both FW and BW (Groups 1 and 2), the mean value of the coefficient of similarity of ES-responses was significantly higher during FW- and BW-period as compared to that during NW- and FW-period (paired *t*-test; Group 1: 69 ± 23 % vs 45 ± 38 %, *P* = 1.1 × 10^−7^; Group 2: 61 ± 32 % vs 41 ± 39 %, *P* = 0.002; Fig. 8F). By contract, in groups of locomotor neurons which contribute to control of FW only (Group 3a) and BW only (Group 3b), as well as in non-modulated neurons the mean values of the coefficient of similarity were similar during NW- and FW-period and during FW- and BW-period (paired *t*-test, *P* ≥ 0.053; Fig. 8F).

**Fig. 9.**
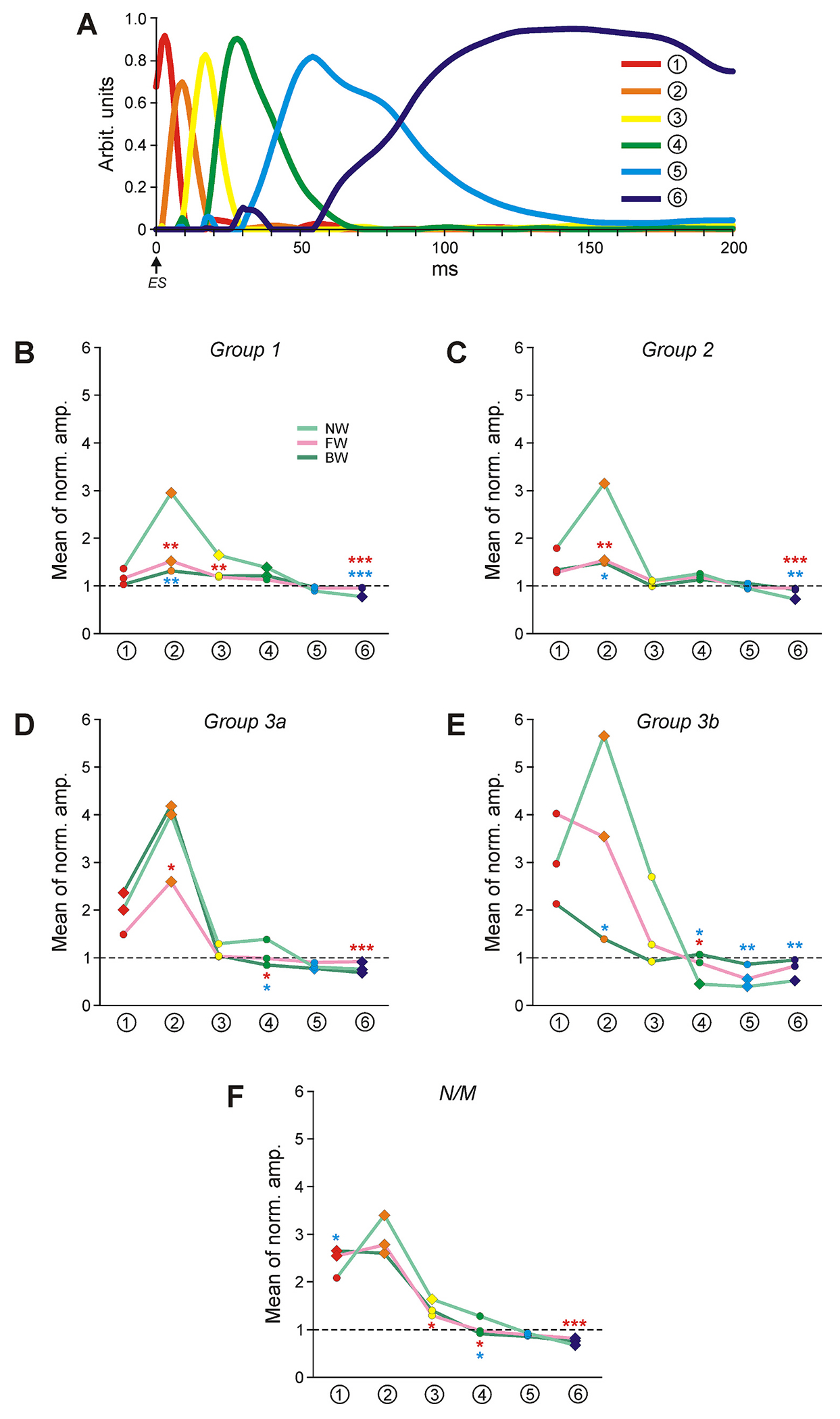
Comparison of relative amplitude of different components of ES-responses in different functional groups of neurons during NW-, FW-, and BW-period. A. Six components of ES responses (##1–6, indicated by numbers in circles; see Methods for details). B-F. Comparison of the amplitudes of each of six components during NW-, FW-, and BW-period in different functional groups of neurons. The amplitude of the component in a particular group of neurons in a specific condition was normalized to their mean frequency in the same condition (dashed line indicates the amplitude level equal to the mean frequency). Red and blue asterisks indicate, respectively, a significant difference of the component value during FW- and BW-periods as compared to that during NW-period. The values of components indicated by diamonds in B-F were different significantly from the value of the mean frequency (from level of 1). Indication of significance level: * *P* < 0.05, ** *P* < 0.01, *** *P* < 0.001. (For interpretation of the references to colour in this figure legend, the reader is referred to the web version of this article.)

To further characterize changes in ES-responses associated with initiation of locomotion, we searched for typical features of ES-responses (see Materials and Methods for the details). Our analysis revealed six components of ES-responses, reflecting inputs to the neuron after each ES stimulus (Fig. 9A). Three earlier components (#1–3) had relatively short latency (up to ~10 ms) and duration (up to ~20 ms). Two later components #4 and #5 had longer latencies (~15 ms and ~ 30 ms, respectively) and duration (~50 ms and ~ 100 ms, respectively). Finally, component #6 had the longest latency and lasted until the next ES stimulus. Most likely, this component reflects the drive from the locomotor CPG rather than a response to an individual ES stimulus. It should be noted that each component can represent both a peak in the ES-response (if its amplitude is higher than the amplitudes of its neighbor components) as well as a trough (if its amplitude is lower than the amplitudes of the neighbor components).

To reveal changes in profiles of ES-responses with transition from NW- to FW- and BW-period, we normalized amplitudes of components ##1–6 of each ES-response to its mean firing frequency (i.e., after normalization, the component amplitude equal to the mean firing frequency became equal to 1). Such normalization neglects the general level of activity but instead focuses on the response profile. The normalized component amplitudes were averaged for different functional groups separately during NW-, FW-, and BW-periods. The results are presented in Fig. 9B–F).

In general, in all groups of neurons during NW-period the profile of ES-response was characterized by high earlier activity (components ##1–3 were above 1) and weaker later activity in the second half of the response (components ##5,6 were below 1). In Groups 1 and 2 (that contribute to control of both FW and BW), amplitudes of the components which were above 1 during NW-period decreased during both FW and BW, while those that were below 1 during NW-period increased (significant changes with transition from NW-period to FW and BW are marked, respectively, by red and blue asterisks in Fig. 9B–F). As a result, the significant (although rather small) difference from 1 remained only for component #2 in Groups 1 and 2 during FW as well as for component #4 in Group 1 during BW (indicated by diamonds in Fig. 9B,C). Thus, due to a decrease in earlier activity (components ##1–4) and an increase in later activity (component #6), on average, the profiles of ES-responses in Groups 1 and 2 during locomotion became flat (all components of the ES-response were close to the average activity level). In the following text, such changes are termed as “flattening” of ES-response.

In Groups 3a and 3b (that contribute, respectively, to control of FW only and BW only) flattening of ES-response was observed only during the locomotor behavior to which the corresponding Group contributed. Thus, in Group 3a, only during FW all averaged components of ES-response changed toward 1 (nevertheless, component #2 remained significantly above 1 and component #6 remained slightly but significantly below 1; Fig. 9D), while in Group 3b similar changes were observed only during BW (all differences of the component averages from 1 were insignificant; Fig. 9E). Note that during the locomotor behavior to which the Groups 3a and 3b do not contribute, the profiles of ES-responses were similar to those observed during NW-period and retained their high peaks of earlier activity and weaker later activity. Thus, in Group 3a, components ##1,2 were above and components ##4,5,6 were below 1 during BW (Fig. 9D), while in Group 3b, components ##1,2,3 were above and components ##4,5,6 were below 1 during FW (Fig. 9E).

Finally, in non-modulated neurons, the profiles of ES-responses during both FW and BW were similar to those observed during NW-period (components ##1,2 were significantly above and component #6 was significantly below 1; Fig. 9F). Thus, in non-modulated neurons, the flattening of ES-response during both FW and BW was absent.

**Fig. 10.**
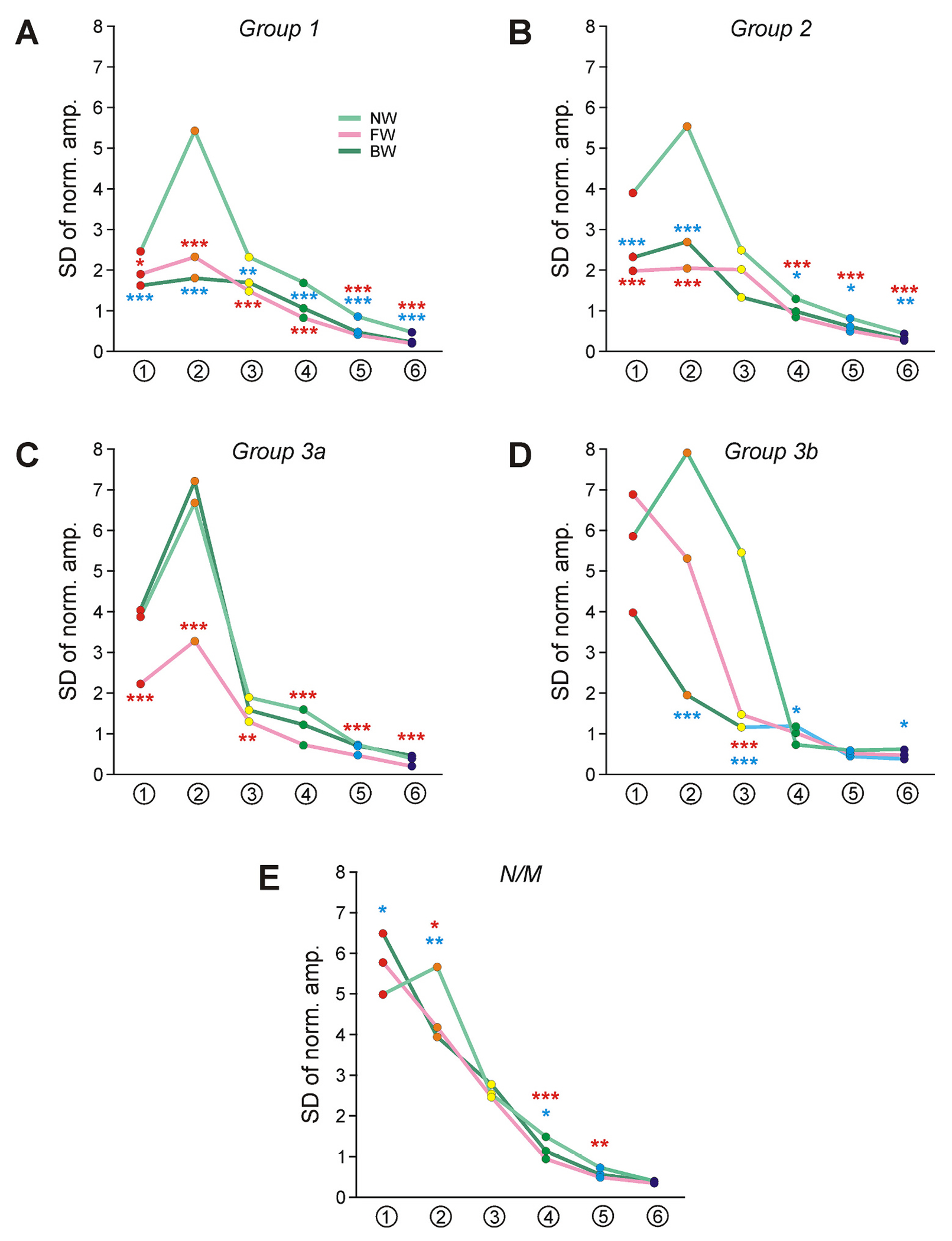
Comparison of variability of the relative amplitude of different components of ES-responses in different functional groups of neurons during NW-, FW-, and BW-period. A-E. SD values of the normalized amplitude of components ##1–6 of ES-responses (shown in Fig. 9) during NW-, FW-, and BW-period in different functional groups. *F*-test was used to compare SDs. Designations as in Fig. 4B-K.

We also found that in Groups 1 and 2, a significant (often severalfold) decrease in SD values of normalized amplitudes of components ##1–6 was observed during FW- and BW-period (except for component #3 of Group 2) as compared with those during NW-period (Fig. 10A,B). By contrast, in Groups 3a and 3b, a significant decrease in SD value of most components was observed only during the locomotor behavior to which the corresponding Group contributed. Thus, in Group 3a, the decrease was observed during FW (Fig. 10C) while in Group 3b it was observed during BW (Fig. 10D). Finally, in non-modulated neurons (Fig. 10E), in some components the value of SD increased (in component #1 during BW-period) and in some components it significantly decreased during locomotion (in component 2 during both FW- and BW-period) as compared with that in NW-period. Thus, in locomotor Groups of neurons, the variability of amplitudes of different components of ES response as compared with those in NW-period was significantly decreased during the type of locomotion to which generation the Group contributed.

## 4. Discussion

In the present study, to reveal effects of ES on the activity of spinal neurons that lead to initiation of locomotion, we compared: (i) the activity of individual neurons at three conditions - in the absence of ES (TM-period), in the initial period of ES (before the first reciprocally coordinated left-right steps were generated, NW-period), and during well-coordinated FW/BW (FW/BW-period); (ii) responses of neurons to individual epidural stimuli (ES-responses) during NW-, FW-, and BW-period. The comparison was done for different functional groups of neurons (locomotor Group 1–3 neurons and non-modulated neurons) as well as for neurons located in the stimulated segment (L5), in segments rostral (L4), and caudal (L6) to stimulation.

### 4.1. Beginning of locomotion is associated with specific changes in activity of individual locomotor neurons rather than with changes in their population activity level

ES activates locomotor mechanisms through large-diameter myelinated sensory fibers of dorsal roots (Gerasimenko et al., 2005; Capogrosso et al., 2013; Moraud et al., 2016; Formento et al., 2018). It was hypothesized that to start locomotion, the activity level of locomotor neurons must be above some necessary minimum. It was suggested that stimulation of sensory afferents leads to gradual building up of neuronal activity and when the activity exceeds the required level, the generation of locomotion begins (Grillner and Zangger, 1979).

Indeed, we found that in each of L4-L6 segments, as well as in each of Groups of locomotor neurons and in non-locomotor neurons, ES strongly activated the overwhelming majority of neurons during NW-period. Since after entering the spinal cord, afferents of dorsal roots bifurcate into ascending and descending branches that send terminals to multiple spinal segments (Edgley and Jankowska, 1987a; Mense and Craig, 1988), it is not surprising that ES similarly affects neurons located in stimulated as well as neighboring segments. Although the large-diameter afferents activate both excitatory and inhibitory neurons (Edgley and Jankowska, 1987b; Bannatyne et al., 2006, 2009; Jankowska et al., 2009; Jankowska and Edgley, 2010), our results demonstrate that the “net effect” of their activation is excitatory.

We expected that the average activity of neurons (and in particular, locomotor neurons) would be lower during gradual building up of neuronal activity in NW-period as compared to that in FW/BW-periods. Surprisingly, we found that transition from NW-period to FW/BW was not accompanied either by substantial changes in the mean frequency of neuronal populations located in each of L4-L6 segments, or by specific changes in the mean frequency of different locomotor Groups and in non-locomotor neurons. Thus, ES-caused activation of the neurons of the locomotor network to an average level similar to that observed during locomotion is not sufficient for initiation of stable locomotor rhythm generation.

However, we found a significant trend in the activity change of individual neurons within functional groups with transition from NW- to FW/BW-period: those neurons that were less active during NW-period tended to increase their activity, while those neurons that were more active during NW-period tended to decrease their activity during locomotion. Thus, generation of stable locomotion is associated with a decrease in heterogenety in the activity level of individual neurons and therefore narrowing the range of individual neurons activity. One can suppose that the neurons with insufficiently high or excessively high activity during NW-period may hamper initiation of locomotor rhythm generation.

### 4.2. Beginning of locomotion is associated with flattening of responses of locomotor neurons to individual ES stimuli

According to their pattern, six Types of ES-responses during NW-period were revealed. Only in 29 % of neurons a sharp excitatory response was observed (Types 1 and 2). Most likely, a part of these responses with shortest latencies were monosynaptic. It was demonstrated that Group 1 afferents monosynaptically affect both excitatory and inhibitory neurons (Edgley and Jankowska, 1987b; Bannatyne et al., 2009; Jankowska et al., 2009). Surprisingly, in almost half of neurons (44 %), the earlier component of the ES-response was inhibitory. One can suppose that some neurons with Types 1 and 2 responses are inhibitory and cause this earlier inhibitory component. ES-responses in most neurons were characterized by sequential waves of excitation (Types 3–5) or inhibition (Type 6). Most likely these waves of excitation/inhibition reflect activation of polysynaptic reverberating circuits. It was suggested earlier that activation of polysynaptic spinal circuities is necessary for activation of locomotor networks by ES (Gerasimenko et al., 2008).

We found that the patterns of ES-responses during FW-period substantially differed from those during NW-period (*K_SIM_* ~ 40–45 %) both in locomotor (Groups 1–3) and in non-modulated neurons. The difference can be caused either by inputs from other neurons of the activated locomotor network or by substantial changes (gating) in the pathways transmitting signals from activated by ES afferents to neurons taking place with the onset of locomotion. Gating of sensory inputs from specific afferents to spinal neurons with the onset of locomotion was demonstrated earlier (Duysens and Pearson, 1980; Conway et al., 1987; Shefchyk et al., 1990; Gossard et al., 1994; Quevedo et al., 1998; McCrea, 1998; Angel et al., 2005; Musienko et al., 2020, 2022). We also found that in Group 1 and Group 2 neurons (that contribute to generation of both FW and BW) the difference in patterns of ES-responses with transition from FW to BW was significantly smaller (*K_SIM_* ~ 60–65 %) as compared to that observed with transition from NW-period to FW. One can suggest that gating of ES-responses in Group 1 and Group 2 neurons is produced by the rhythm generating part of the locomotor network that operates similarly during both FW and BW and generates the vertical component of the step (Musienko et al., 2012, 2022). By contrast, in Group 3a and Group 3b neurons (contributing to generation of FW only and BW only, respectively) as well as in non-modulated neurons, the changes in the pattern of ES-response with transition from FW to BW were similar to those observed with transition from NW-period to FW. Most likely, gating of ES-responses in these functional groups is caused by different parts of locomotor network that operate during FW only and BW only and control the horizontal component of the FW and BW step, respectively (Musienko et al., 2012, 2022).

**Fig. 11.**
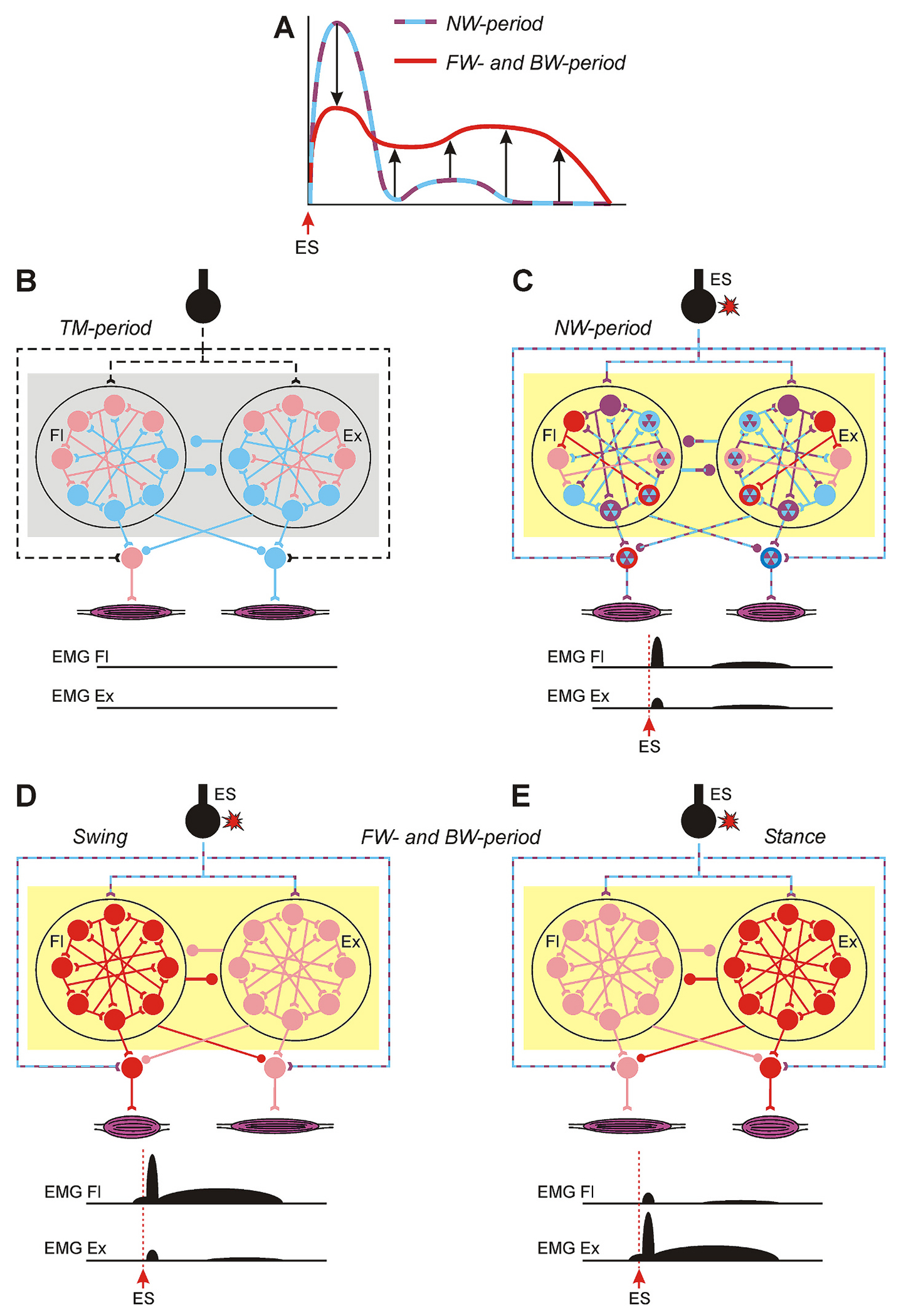
Summary of results. A. Schematic changes in ES-responses with transition from NW-period (dashed purple/blue line) to FW-/BW-period (red line). B-E. Hypothetical spinal circuitry affected by ES is shown in three conditions: during TM-period (B), during NW-period (C), as well as during FW/BW-period when it generates swing (D) and stance (E) phases of the locomotor cycle. In each condition, effects produced by the network on the activity of flexor and extensor muscles (EMG Fl and EMG Ex) are shown schematically. Gray background indicates a condition in which the average activity of locomotor neurons is low, while yellow background indicates a condition in which the average activity of locomotor neurons is similar to that observed during locomotion. Designations: neurons within circles belong to flexor and extensor half-centers of the locomotor network (Fl and Ex, respectively). The neurons outside the circles are motoneurons. Open triangles show excitatory synapses, filled circles – inhibitory synapses. Inactive neurons, neurons with very weak and with extremely high activity (which are out of the locomotor range) are indicated, respectively, by blue, pink, and purple outline as well as their connections are marked by blue, pink and purple, respectively. Neurons with large amplitude peaks and troughs in ES-responses are indicated by purple/blue filled circles and their connections are indicated by dashed (purple/blue) lines. Neurons with activity within the locomotor range and flat ES-responses as well as their connections are indicated by red. In C, higher level of excitability of flexor motoneurons and lower level of excitability of extensor motoneurons are marked by red and blue outline, respectively. See explanations in the text. (For interpretation of the references to colour in this figure legend, the reader is referred to the web version of this article.)

It is well documented that sensory feedback from the limbs transmitted by group 1a and 1b afferents assists in swing/stance transition, and their stimulation in an inappropriate part of the locomotor cycle can disrupt/reset/entrain the locomotor rhythm (Grillner and Rossignol, 1978; Duysens and Pearson, 1980; Pearson et al., 1992, 1998; Hiebert et al., 1996). One of the striking features of ES is that epidural stimuli that strongly activate these afferents do not disrupt/reset/entrain the locomotor rhythm. We demonstrated that with the onset of locomotion, the ES-responses in locomotor neurons which contribute to generation of particular mode of locomotion (FW or BW) became flatter (due to a decrease in high amplitude components and increase in low amplitude components of the ES-response, Fig. 11A), as well as their variability decreased. Thus, most likely the absence of strong sharp components in ES-responses of locomotor neurons during locomotion explains the fact that ES does not entrain/disrupt/reset the locomotor rhythm. In line with this, in neurons not involved in generation of stepping (non-locomotor neurons, and also Group 3a during BW and Group 3b during FW) flattening and decrease in variability of ES-responses were absent.

### 4.3. Hypothesis about mechanism underlying induction of locomotion by ES

Results obtained in the present study are summarized in Fig. 11B–E. We found that while before ES the activity of locomotor neurons was very weak or absent (indicated by gray background in Fig. 11B), most locomotor neurons were activated during NW-period (Fig. 11C). Although the mean level of the activity of population of locomotor network neurons was similar to that observed during locomotion (indicated by yellow background in Fig. 11C,D), the activities of individual locomotor neurons as well as their ES-responses significantly differed from those observed during locomotion. The heterogeneity in the activity level of individual locomotor neurons was very large (indicated by different colors outline of neuronal cell bodies in Fig. 11C), and ES-responses in many neurons were characterized by high peaks and deep troughs (indicated by purple/blue fill of neuronal cell bodies in Fig. 11C). One can assume that these factors hampered the necessary coordination of locomotor neurons required for generation of the locomotor pattern. Instead, mES-responses (responses in muscles to single ES stimuli) with more consistent earlier [presumably monosynaptic (Gerasimenko et al., 2003, 2005, 2006; Lavrov et al., 2006, 2008; Musienko et al., 2009)] and less consistent later (polysynaptic) components were observed. Stronger mES-responses in flexor muscles as compared with those in extensor ones can be explained by a higher level of excitability of flexor motoneurons caused by activation of flexor stretch receptor afferents due to extension of limbs during NW-period caused by treadmill belt motion.

We found that transition from NW- to FW/BW-period was associated with an increase and decrease in activity of individual locomotor neurons with low and high activity, respectively, as well as with flattening and a decrease in variability of their ES-responses (indicated by homogeneous fill of neuronal cell bodies in Fig. 11D,E). The mechanisms underlying these changes are not clear. It was suggested that activation of polysynaptic pathways underlying appearance of the later component of ES-response is necessary for initiation of ES-caused locomotion (Gerasimenko et al., 2008). One can assume that these activated by ES polysynaptic pathways include Group 1 interneurons (presumably forming swing and stance half-centers, respectively, Musienko et al., 2022) and long-latency components of ES responses (e.g., components ##5,6) of some of these Group 1 neurons are responsible for generation of the later component of the mES-responses (Fig. 11C). Most probably during NW-period, within each of the half-centers, a part of activated Group 1 neurons (that had activity in the locomotor range and flat ES-responses, indicated by red in Fig. 11C) were recruited into rhythm generation. These small rhythm generating networks started to gate pathways mediating ES-responses of other locomotor neurons. This gating resulted in a change of their general activity toward the locomotor range and flattening of the ES-responses. That caused gradual recruitment of these neurons into rhythm generation, finally leading to coordinated activity of the two half-centers and generation of locomotor movements. Alternating activity in swing and stance half-centers during locomotion led to phase-dependent modulation of earlier component of mES-response (Fig. 11D,E).

To conclude, in the present study, for the first time, effects of ES on the activity of individual spinal neurons during the initial period of ES as well as during well-coordinated FW and BW were analyzed. We demonstrated that the onset of ES causes activation of the overwhelming majority of spinal neurons. Although on the population level the activity of spinal neurons in the initial period of ES and during FW/BW was similar, the activity of individual neurons within each functional population as well as their responses to individual epidural stimuli at these two conditions were substantially different. With the onset of a specific mode of locomotion (FW or BW) the range of activities of individual neurons contributing to its generation was narrowed, as well as ES-responses of individual neurons were significantly flattened. We suggest that these changes allow to maintain a high level of excitability of locomotor network without distortion of the locomotor rhythm. The obtained results provide new insights into neuronal mechanism underlying activation of locomotor network by ES.

## Figures and Tables

**Table 1:** Neuronal database.

	Cat #1 *(L4)*	Cat #2 *(L5)*	Cat #3 *(L6)*	Cat #4 *(L6)*	Total
*Group 1*	14 (25 %)	24 (39 %)	7 (39 %)	56 (31 %)	101 (32 %)
*Group 2*	14 (25 %)	7 (11 %)	6 (33 %)	38 (21 %)	65 (21 %)
*Group 3a*	12 (21 %)	19 (31 %)	5 (28 %)	24 (13 %)	60 (19 %)
*Group 3b*	9 (16 %)	2 (3 %)	0	10 (6 %)	21 (7 %)
*N/M*	7 (13 %)	10 (16 %)	0	51 (29 %)	68 (21 %)
*Total*	56 (100 %)	62 (100 %)	18 (100 %)	179 (100 %)	315 (100 %)

Number of neurons in Groups recorded in individual animals. Percentage of neurons constituting individual Groups in individual animals as well as in all animals (*Total*) is shown in parentheses.

**Table 2:** Effects of the ES onset on activity of neurons located in different segments and on activity of neurons from different functional groups.

	ΔF (Hz)	Significance
*L4*	10.1 ± 11.2	*P* = 1.1 × 10^−8^
*L5*	14.1 ± 20.3	*P* = 8.4 × 10^−7^
*L6*	14.6 ± 16.7	*P* = 6.2 × 10^−26^
*Group 1*	14.0 ± 18.4	*P* = 1.3 × 10^−11^
*Group 2*	15.3 ± 13.8	*P* = 7.5 × 10^−13^
*Group 3a*	13.0 ± 16.1	*P* = 5.1 × 10^−8^
*Group 3b*	9.2 ± 12.0	*P* = 2.0 × 10^−3^
*N/M*	13.7 ± 18.5	*P* = 5.4 × 10^−8^

*ΔF* is the average value of the difference between activity of neurons during NW-period and TM-period for neuronal populations located in L4, L5, and L6 segments, as well as for Groups 1, 2, 3a, 3b, and non-modulated neurons (N/M). Paired *t*-test was used to reveal the significance of the frequency increase.

## Data Availability

The data that support the findings of this study are available from the corresponding author upon request.
